# Photodynamic Therapy and Adaptive Immunity Induced by Reactive Oxygen Species: Recent Reports

**DOI:** 10.3390/cancers16050967

**Published:** 2024-02-28

**Authors:** David Aebisher, Paweł Woźnicki, Dorota Bartusik-Aebisher

**Affiliations:** 1 Department of Photomedicine and Physical Chemistry, Medical College of the University of Rzeszów, 35-959 Rzeszów, Poland; 2 Students English Division Science Club, Medical College of the University of Rzeszów, 35-959 Rzeszów, Poland; pw118616@stud.ur.edu.pl; 3 Department of Biochemistry and General Chemistry, Medical College of the University of Rzeszów, 35-959 Rzeszów, Poland; dbartusikaebisher@ur.edu.pl

**Keywords:** cancer, immunity, reactive oxygen species

## Abstract

**Simple Summary:**

Photodynamic therapy (PDT) is a cancer treatment that uses photogenerated reactive oxygen species (ROS) to damage target cells. The unique mechanism of action of PDT involves the systemic or local administration of a photosensitizing compound (photosensitizer), which is then activated by light of a specific energy. PDT induces a very strong local inflammatory response. A number of adaptive mechanisms are induced within the tumor, related to increased amino acid metabolism and damage to lymphatic vessels. In this review, we are describing the adaptive immune response induced by ROS and generated by PDT.

**Abstract:**

Cancer is one of the most significant causes of death worldwide. Despite the rapid development of modern forms of therapy, results are still unsatisfactory. The prognosis is further worsened by the ability of cancer cells to metastasize. Thus, more effective forms of therapy, such as photodynamic therapy, are constantly being developed. The photodynamic therapeutic regimen involves administering a photosensitizer that selectively accumulates in tumor cells or is present in tumor vasculature prior to irradiation with light at a wavelength corresponding to the photosensitizer absorbance, leading to the generation of reactive oxygen species. Reactive oxygen species are responsible for the direct and indirect destruction of cancer cells. Photodynamically induced local inflammation has been shown to have the ability to activate an adaptive immune system response resulting in the destruction of tumor lesions and the creation of an immune memory. This paper focuses on presenting the latest scientific reports on the specific immune response activated by photodynamic therapy. We present newly discovered mechanisms for the induction of the adaptive response by analyzing its various stages, and the possible difficulties in generating it. We also present the results of research over the past 10 years that have focused on improving the immunological efficacy of photodynamic therapy for improved cancer therapy.

## 1. Introduction

Cancers are one of the most significant causes of death worldwide. Their etiology is multifactorial and results from the complex interactions of many risk factors, both environmental and genetic [1,2]. Despite increasingly well-developed forms of treatment, such as chemotherapy and radiation therapy, prognosis and mortality rates among cancer patients remain poor. Metastasis and recurrence are two main problems that impede progress in cancer treatment [2]. Therapeutic solutions are hampered by the fact that cancer cells benefit from a dysfunctional immune environment incapable of triggering an anti-tumor response [3]. Therefore, new forms of therapy capable of destroying the primary tumor and residual lesions are constantly being sought [2]. One mode of treatment that continues to be developed and studied is photodynamic therapy (PDT) [2,3,4,5]. Currently, this method has been approved for the treatment of head and neck cancer, esophageal cancer, pancreatic cancer, prostate cancer, and esophageal squamous cell carcinoma [6,7]. In addition, it is widely used in dermatology for the eradication of precancerous and cancerous lesions [8]. Photodynamic therapy involves administering a photosensitizer (PS) that either selectively accumulates in the tumor tissue (tissue-based PS) or circulates in the tumor vasculature (vascular PS) prior to irradiation with light at a wavelength corresponding to PS absorbance, leading to the induction of reactive oxygen species (ROS), such as singlet oxygen, hydroxyl radical, superoxide ion, hydrogen peroxide and others [9,10,11,12,13,14]. Tumor destruction occurs through direct killing of tumor cells, closure of tumor vessels, and induction of local inflammatory responses with activation of the immune system [3,5,9,10,11,12,13,15,16,17]. Activation of the innate immune response is essential for the subsequent induction of the adaptive immune system response [10]. This process involves the release of damage-associated molecular patterns (DAMPs), the cross-presentation of tumor antigens by dendritic cells (DCs) to T lymphocytes, and their specific anti-cancer response [18,19,20,21,22,23,24]. Moreover, T lymphocytes can form an immune memory that provides long-term protection against cancer recurrence [25]. The role of reactive oxygen species in these processes is complex. On the one hand, they are critical mediators of the formation of innate and acquired immunity [26]. Furthermore, they are essential for inducing immunosuppression in the tumor microenvironment (TME) [27]. For PDT to be effective in cancer therapy, it should meet several criteria: PS should selectively accumulate in tumors, have low toxicity in the dark, and be simple to synthesize, and PDT itself should have the ability to induce immunogenic cell death (ICD) [26]. In addition, the effect of PDT can be enhanced by association with other forms of therapy, such as checkpoint-blocking immunotherapy, chemotherapy, radiotherapy, and enzyme inhibitors [2,4]. Combination therapy can reduce the necessary drug dose, significantly improve patients’ quality of life, reduce side effects associated with a single therapy, and increase anti-tumor activity, thus providing a significant survival benefit [2]. The strength of PDT is that it is characterized by minimal invasiveness, spatiotemporal selectivity, and few side effects [6,14]. However, PDT is still not a regular treatment option for cancer, which may be due to limitations of PDT, such as the accessibility of the tumor site and the depth of light penetration through tissue needed to excite the photosensitizer, ineffective induction of cancer cell death, tumor resistance to therapy, hydrophobicity, and PDT-induced photosensitization of the skin [6,10]. Obstacles to the adaptive immune response result from the complex interactions of immune cells, tumor cells, immunosuppressive cells, and the tumor microenvironment (TME) [27]. In this paper, we focus on presenting evidence of PDT’s ability to activate a specific immune response, along with recent reports on its mechanism. We also analyze the reasons for the possible failure of PDT in this challenge and we present the latest reports from recent years focusing on improving the immunological efficacy of PDT for advancing cancer therapy.

## 2. Materials and Methods

**Figure 1:**
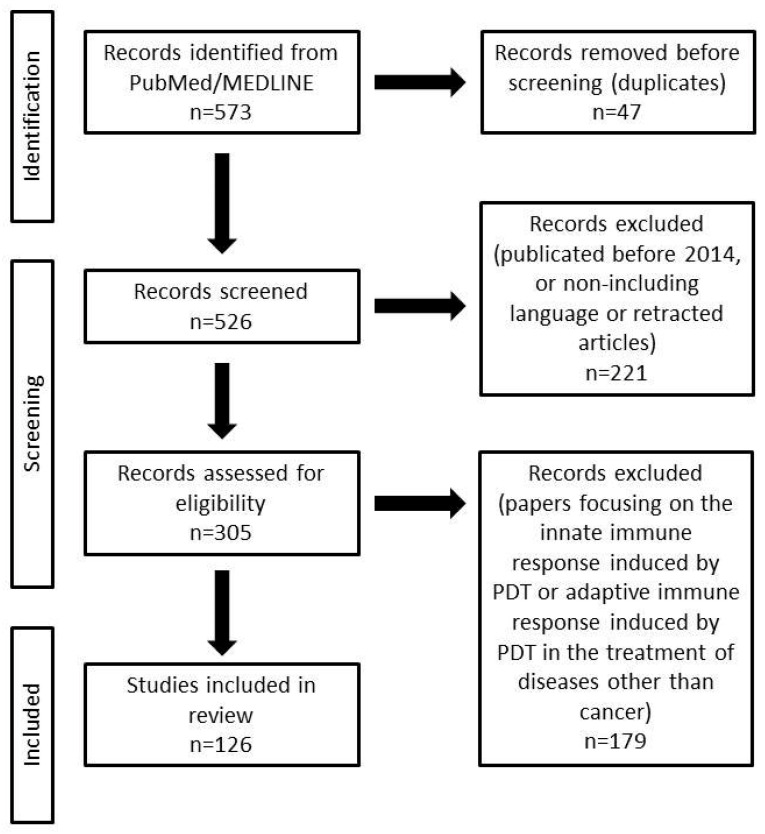
PRISMA flow diagram of the studies included.

A literature search that focused on papers describing the adaptive immune response induced by ROS generated by PDT for the treatment of malignant tumors was conducted using the PubMed/MEDLINE database. The following terms were searched for: “Adaptive immunity AND PDT”, “Adaptive immunity AND reactive oxygen species AND PDT”, “Adaptive immunity AND reactive oxygen species AND cancer”. A total of 573 articles were located. Both in vivo and in vitro studies, research papers and review articles were qualified. After excluding duplicates, 526 papers were qualified for review. To describe the latest knowledge, articles written before 2014 were excluded. In addition, taking into account the language criterion, and after excluding retracted papers, 305 papers were qualified for review. Included in the review are papers that characterize the mechanism of induction of adaptive immunity by PDT and possible reasons for the failure of this therapy. In addition, papers describing recent reports on the immune efficacy of PDT and the establishment of effective cancer therapy were also included (Table 1). Finally, 126 articles were used for this study (Figure 1).

## 3. Generation of Reactive Oxygen Species

The mechanism of reactive oxygen species induced by PDT is well-described and well-established. In short, after selective accumulation in tumor cells, the photosensitizer is locally activated by irradiating it with light at a wavelength corresponding to the absorbance of the PS [9,10,11]. This process excites the PS from the ground state to the excited singlet state followed by intersystem crossing to the excited triplet state [14]. Then, the PS in the excited triplet state transfers its energy to molecular oxygen, leading to the formation of highly reactive singlet oxygen or undergoes electron transfer reactions with molecules to form free radicals such as hydroxyl radical, superoxide ion, or hydrogen peroxide [10,11,14,28]. Reactive oxygen species are characterized by their high reactivity, thus their effective range is less than 0.02 µm [16]. These molecules are responsible for oxidative damage to proteins, lipids, and other cell components, ultimately leading to cell death [11,14,28].

## 4. Activation of the Immune Response

**Figure 2:**
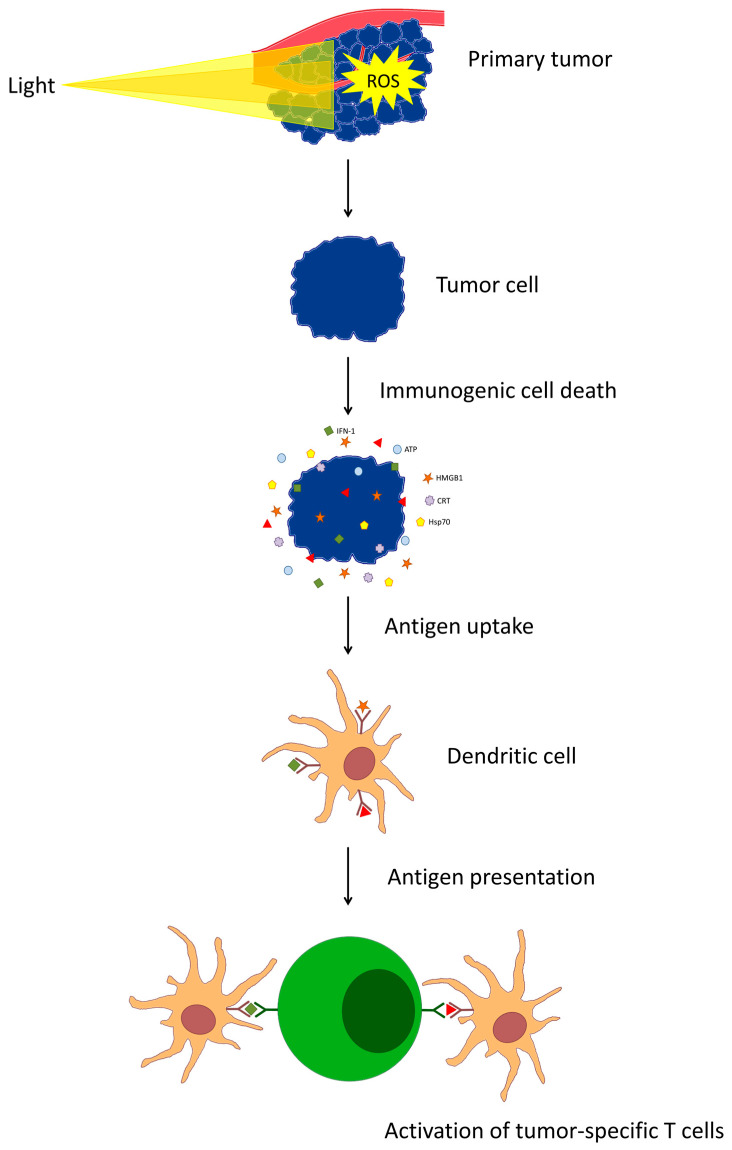
Mechanism of T cell activation involving dendritic cells after PDT.

The precise mechanism of PDT-induced activation of the immune response has not yet been well defined [29]. Reactive oxygen species generated by photodynamic action damage intracellular organelles leading to apoptosis, necrosis, pyroptosis, and immunogenic cell death [9,10,12,20,21,22,30]. Tumor cell death is responsible for the release of molecular patterns associated with damage and is the first and necessary step in the activation of the immune response induced by PDT [20,21,22]. The occurrence of this phenomenon depends on the photosensitizer used [22]. Recent reports include evidence that immune responses are induced by 5-aminolevulinic acid (prodrug producing endogenous protoporphyrin IX), tetracyanetetra(aryl)porphyrazines, zinc phthalocyanine (ZnPc), rhenium(I) compounds, and porphyrin lipoproteins [21,30,31,32,33,34,35]. DAMPs released by PDT include calreticulin (CRT), HSP70, high mobility group box 1 (HMGB1), interferon-1 (IFN-1), and ATP [21,31,33,36]. These molecules trigger a strong inflammatory response and send a signal to the innate immune system, resulting in the activation of certain cell types (including NK cells) and the release of additional pro-inflammatory factors and cytokines [7,10,13,37,38,39,40]. Activation of the innate immune response is essential for the subsequent induction of the adaptive response and the formation of long-term immune memory generated by PDT [10,21,22]. However, the main function of DAMP in the induction of an adaptive response is to promote the activation and uptake of tumor-associated antigens (TAA) by antigen-presenting cells (APCs) [20,31,34]. DAMPs are crucial in the induction of dendritic cell maturation, both phenotypically, involving an increase in surface expression of MHC-II, CD80, and CD86, and functionally, involving increased secretion of interferon-γ (IFN-γ) and IL-12 [31]. An increase in MHC II expression was observed after PDT [35]. DCs endocytose tumor cell-derived microparticles (T-MPs) containing tumor antigen profiles into lysosomes [41]. Then, through activation of the NADPH oxidase 2 (NOX_2_) complex, there is an increase in the concentration of ROS and lysosomal pH from 5.0 to 8.5, lipid peroxidation, and a membrane-damaging chain reaction, which promotes the leakage of antigens from endosomes and the formation of peptide complexes of tumor antigens and MHC class I [41,42,43,44]. Tumor lysates formed by photodynamic therapy-induced cell damage and death may be responsible for the increased ability of dendritic cells to present antigens [32]. Then, these cells migrate to the lymph nodes draining the tumor, where they present the foreign antigen in MHC-I molecules to cytotoxic T lymphocytes in a process called cross-presentation [24,43,45,46,47,48]. This stage, which is necessary to initiate a CD8+ T-cell response against tumor cells, is also enhanced by PDT [24,35,45]. After antigenic stimulation, T cells undergo metabolic changes for proliferation and differentiation, and the direction of lineage selection of these cells likely depends on glutamine catabolism driving de novo glutathione synthesis [23]. CD8+ T lymphocytes induce both an immune response, ultimately eliminating distant tumors at the same time as primary tumors, as well as playing a fundamental role in the formation of long-term immune memory [14,30,49,50,51,52,53,54]. Photodynamic therapy has also been shown to lead to a change in the proportion of CD4+ T-cell subsets in the spleen and an increase in the frequency of CD8+ T-cells in the distal, non-irradiated lymph nodes draining the tumor [35]. However, the adaptive immune response also requires the participation of memory CD8+ T cells and B lymphocytes [51,55]. It has been observed that antibodies produced by photodynamic tumor therapy targeting tumor cells through the Fc region induce engulfment of these cells by macrophages as well as stimulate neutrophil antibody-dependent cytotoxicity [52] [16,56]. Neutrophils also have the ability to modulate the adaptive response by influencing dendritic cells and lymphocytes either directly or through cytokines, highlighting the complex effects of PDT on adaptive immunity mechanisms [57,58]. The mechanism of T cell activation involving dendritic cells after PDT is presented in Figure 2.

## 5. Problems

The tumor microenvironment can cause resistance to PDT and other forms of therapy by weakening anti-tumor immune responses, as is well established in the literature [59,60,61,62,63,64,65,66,67,68]. The TME is complex and constantly changing, with the effect occurring through numerous mechanisms, such as the creation of an immunosuppressive environment around the tumor, or the presence of cells with a suppressor function against functional immune cells [60,61,62,63,64,65,66,67,68,69,70,71]. The TME is made up of the stroma, extracellular matrix (ECM), blood and lymphatic vessels, nerve, and immune cells [66].

### 5.1. Soluble Factors and Extracellular Matrix

In particular, cytokines, metabolites, and ROS are responsible for the immunosuppressive properties of the TME and overcoming PDT-induced immune surveillance, but the extracellular matrix also has this ability [61,62,63,64,65,66,67,68,72,73,74,75,76]. Chronic inflammation occurring in the TME is characterized by the continuous release of multiple inflammatory factors, which have been shown to have the ability to inhibit the function of effector immune cells [62,72]. Reactive oxygen species present in high concentrations in the TME can inhibit PDT-activated T cells by inducing oxidative stress [67,68,75]. The TME destabilizes the loading and dispatch of newly synthesized proteins, inducing endoplasmic reticulum stress in effector lymphocytes and leading to rapid loss of their function [77,78]. Via the FcγRIIb-p38MAPK-ROS signaling pathway, C-reactive protein (CRP) can also attenuate the anti-tumor response [72]. Similar effects are produced by lactic acid produced by tumor cells in the Warburg effect pathway, further inhibiting T-lymphocyte activation [63,74]. Nitric oxide at low concentrations can be the reason for immunosuppressive phenotypes through metabolic reprogramming [76]. Tumor cell-derived microparticles have also been shown to induce the immunosuppressive M2 phenotype of macrophages [79,80]. Other immunosuppressive effects of TME are insufficient availability of nutrients for immune cells, worsening their resistance to stress [66,73]. In addition, CD8+ T-cell inefficiency may be due to impaired glycosylation induction through deficiency of NF-κB-inducing kinase (NIK) in the tumor microenvironment [81]. Hypoxia in the TME not only reduces PDT’s ability to produce ROS but also induces immunosuppression by directly affecting immune system effector cells [64,66,76]. Moreover, it promotes the formation of DNA damage that can have a mutagenic effect and thus allow resistant cancer cells to survive [76]. The interaction between the cells and the extracellular matrix (ECM) induces the release of soluble factors responsible for remodeling the ECM and evading the immune response. Other immunodepressive aspects of the TME include the presence of tumor cell-derived exosomes, circulating deregulated microRNAs, and abnormal mechanical forces [66].

### 5.2. Cells

Cells found in the TME with suppressor functions include myeloid-derived suppressor cells (MDSCs), regulatory T cells (Treg), granulocytes, M2 macrophages, and others [59,61,79,80,82,83,84,85,86,87,88,89,90,91,92,93]. Hypoxic tumors are characterized by increased numbers of these cells as well as decreased infiltration and activation of cytotoxic T cells [94]. In addition, it weakens the anti-tumor effect of PDT.

#### 5.2.1. Myeloid-Derived Suppressor Cells

Myeloid-derived suppressor cells (MDSCs) are immature cells that play a central role in suppressing the anti-tumor immune response [62,85,88,95,96]. Understanding their multifaceted immunosuppressive effects may contribute to understanding the failure of PDT to activate the adaptive immune response. Myeloid-derived suppressor cell expansion was found to be correlated with the advanced stage of the disease, and the incidence of MDSCs was associated with a poor prognosis [95,97]. There are two main populations of MDSCs: monocytic (Mo-MDSC) with a Ly6G-Ly6ChighGr-1intCD11b+ phenotype, and granulocytic or polymorphonuclear (G-MDSC, PMN-MDSC) with a Ly6GhighLy6ClowGr1highCD11b+ phenotype [27,88,97]. It has been observed that the number of Mo-MDSCs is higher in cancer patients than in healthy individuals [87]. Myeloid-derived suppressor cells are formed by altering normal myelopoiesis and converting healthy myeloid cells [62]. Recent reports indicate that extracellular vesicles secreted by the tumor play a key role in this process [62]. Recently, it was also shown that through the effect of IL-18 on the differentiation of CD11b(-) bone marrow progenitor cells, there is an increase in the number of Mo-MDSCs and that the absolute number of G-MDSCs does not change at the same time [98]. Moreover, strong activation of these cells can also be induced by trans-membrane TNF-α (tmTNF-α), which, acting as a ligand for tumor necrosis factor receptor 2 (TNFR2), promotes the secretion of NO, ROS, IL-10, and tumor necrosis factor β (TGF-β) [84]. Subsequently, these cells are recruited by chemotaxis into the inflamed tissues, where they proliferate [99]. Both of these processes are initiated and stimulated by numerous soluble inflammatory mediators [62,98,99]. Lysosomal acid lipase deficiency has also been shown to be responsible for MDSC infiltration, but the exact mechanism of this phenomenon is unknown [100]. Macrophage migration-stimulating factor may have the same effect [101]. The mechanisms by which MDSCs exert their immunodepressive effects are characterized by great complexity [27,96,97,99,102]. Myeloid-derived suppressor cells are a major mediator of the induction of suppression of tumor-reactive T cells, inhibit NK cells, as well as stimulate the formation of regulatory T cells, thus inhibiting the PDT-induced adaptive immune response through several pathways [27,86,87,96,99] [102]. It was observed that G-MDSCs more potently inhibit activated T cells through their ability to attenuate proliferation and expression of effector molecules compared to Mo-MDSCs [27]. Oxygen free radicals, including ROS, NO, peroxynitrite (PNT) arginase, indoleamine 2,3-dioxygenase, prostaglandins, the pro-inflammatory heterodimer S100A8/9 and cytokines such as granulocyte–macrophage colony-stimulating factor (GM-CSF), interleukin 10 (IL-10) and transforming TGF-β have been shown to be particularly effective in inhibiting the cellular antitumor response [27,60,86,87,96,97,99,102]. This fact is especially interesting given that NO formation can be stimulated by PDT [11]. The molecules involved in T-lymphocyte immunodepression vary depending on the MDSC phenotype expressing them, e.g., G-MDSCs use peroxynitrite while Mo-MDSCs use NO [27]. In addition, their expression can be correlated with clinical progression, as observed for GM-CSF [60]. Despite the environmental concentration of ROS also elevated by PDT, have the ability to survive due to NF erythroid 2-related factor 2 (Nrf2), which is a transcriptional factor that regulates genes that alleviate oxidative stress [102]. Myeloid-derived suppressor cells also stimulate the activity of immunosuppressive regulatory T cells. Moreover, MDSCs also induce immunosuppression by disrupting energy metabolism and tissue proteostasis [99]. 

#### 5.2.2. Other Immunosuppressive Cells

Another cell type with a well-established and crucial suppressive effect on the anti-tumor immune response is regulatory T cells [59,82,83]. Their formation is stimulated by MDSCs, but TNFR2 is also involved in this process [84,99]. These cells provide a favorable environment for tumor growth by inhibiting the activation and expansion of tumor antigen-specific effector T cells [82]. Reactive oxygen species-stimulated SUMO1/sentrin/SMT3 specific peptidase 3 (SENP3) accumulation has been shown to be responsible for this immunosuppressive effect [69]. At the same time, it has been established that Glutathione peroxidase 4 (GPX4) plays a key role in protecting regulatory T cells from excessive ROS and ROS-induced lipid peroxidation and ferroptosis [83]. The suppressor functions of regulatory T cells are negatively affected by the expression of PD-1 by these cells [103].

In addition, tumor-associated macrophages, neutrophils, eosinophils, B lymphocytes, and activated normal-density granulocytes (NDG) are also involved in immune suppression [59,79,80,89,96,104]. One study demonstrated the ability of PDT to reprogram immunosuppressive tumor-associated macrophages into anti-tumor M1 macrophages [92]. Recent evidence suggests that neutrophils have the ability to suppress the immune system through several pathways. First, they have the ability to suppress T-cell activation and proliferation through complement-dependent adhesion to their membranes, troglodytosis, and impairment of nuclear factor of activated T-cells (NFAT) translocation, IL-2 production, glucose uptake, mitochondrial function, and activation of mammalian target of rapamycin (mTOR) [90]. In addition, degrading chemokines via extracellular neutrophil traps (NETs) have the ability to completely inhibit the chemotaxis of activated T cells into the tumor [105]. Finally, they weaken the function of the immune system with reactive oxygen species, nitrite, and H_2_O_2_, molecules that are also produced during PDT [91,105]. They also observed PD-L1 expression on their surface and the ability to induce PD-1 on CD8+ T cells [91]. Activated normal-density granulocytes have the ability to inhibit T-cell responses in a manner similar to MDSCs, and this effect is dependent on ROS production [89].

### 5.3. Other Immunosuppressive Factors

The heterogeneity and low immunogenicity of the tumor may lead to a poor antitumor response [106,107,108]. Avoidance of the immune response may also be caused by MHC-I deficiency [109]. Depletion of TCD8+ lymphocytes may be another reason for the insufficient immune system response to cure the tumor. It has been observed that phosphatase of activated cells 1 (PAC1) is selectively up-regulated in these cells, leading to a loss of their proliferative and effector capacity [110]. The altered expression of proline dehydrogenase (PRODH) in tumor tissues is also responsible for reduced T-cell infiltration [111]. It was also recently found that elevated expression of the CD47 molecule on tumor cells is characterized by reduced removal by phagocytes expressing the CD47 counter-receptor SIRPα [112]. Tumor survival may also be led by the presence of cancer stem cells resistant to the immune response [113].

## 6. Latest Reports

Over the past 10 years, a significant number of studies have been conducted, the results of which can significantly contribute to enhancing the effectiveness of PDT, introducing it in the clinic, and establishing an effective form of anti-cancer therapy capable of curing primary tumors and metastases. All the described studies were conducted in vivo on tumor-bearing mouse models. By providing a representation of the body as a whole, they allow for a multifaceted analysis of the impact of therapy. Mouse models of cancer can help improve our understanding of the mechanisms of pathology and the effectiveness of therapy, but they do not always capture the full range of phenomena observed in humans. Despite the similarities, however, they can differ significantly. This problem may be a particularly critical point of the research described in the paper, which is due to differences in the functioning of the mouse and human immune systems. Another drawback is that these studies were conducted using a small number of cancerous cell lines, making it impossible to confidently relate their results in the context of therapies for other cancers. In order to confirm the effectiveness of the proposed therapies, clinical trials testing these strategies are needed in the future.

As mentioned earlier, one of the immunological effects of PDT is the increased activation of T lymphocytes, responsible for tumor destruction. A continuing problem, however, is the lack of the most optimal therapeutic regimen with the greatest efficacy. Shams M et al. showed that increased T-lymphocyte activation, reduced tumor growth, and metastasis were characterized by a treatment regimen that included immune-enhancing PDT followed 10 days later by treatment with a regimen that controlled tumor growth. This effect did not depend on the photosensitizer used [114]. These results are particularly important because they point to a specific regimen that can be used for future therapy in humans. Although the final effect did not differ significantly, the conclusions were drawn based only on the study of tumors of two cell lines: Colo26HA and 4T1, which may affect the efficacy achieved. Zhang et al. showed that mice cured of CT26 colon cancer using vascular-targeted vascular-PDT (VPDT) showed varying degrees of resistance to attack by other types of mouse tumor cells, such as 4T1 and EMT6 breast tumor cells [52]. This study points to another therapeutic regimen of PDT effective in activating the systemic immune response. Moreover, it demonstrates that PDT produces this effect not only by inducing direct cancer cell death but also indirectly through vascular damage. In addition, the anti-tumor immune effect of PDT can be enhanced by epigenetic modification of P1A tumor antigen levels via 5-aza-2′-deoxycytidine (5-aza-dC), as Wachowska et al. found in their study [37]. This study shows that a well-designed chemotherapy regimen using 5-aza-dC restores and enhances tumor-associated antigen and MHC class I expression levels in a set of mouse tumors, and the combination with PDT leads to the development of an immune response and long-term survival. A limitation of implementing this combination, however, may be that epigenetic drugs are not selective and may restore oncogene expression accelerating tumor progression. As mentioned earlier, an important problem in PDT cancer therapy is the immunosuppressive effect of TME. Oh DS. et al. by intratumor administration of PS Chlorin e6-conjugated anti-CD25 antibodies achieved a reduction in the number of tumor-associated regulatory T cells, increased IFN-γ expression, and infiltration of effector T cells [82]. However, this study only focused on increasing anti-tumor response at the treatment site, so to confirm the validity of this method, further studies are needed to determine whether this strategy can also inhibit the growth of metastatic tumors at other locations. This study only focused on increasing anti-tumor response at the treatment site, so to confirm the validity of this method, further studies are needed to determine whether this strategy can also inhibit the growth of metastatic tumors at other locations. This method can increase the effectiveness of PDT because it works preferentially in areas of the tumor where hypoxia is more severe. The hypoxia in TME was completely overcome by Su et al. who, by combining a rhenium (I) photosensitizer with carbonic anhydrase IX, created a molecule that effectively stimulated pyroptosis, ensuring full activation of the adaptive immune response in vivo and elimination of metastasis [115]. Of particular interest seems to be the association of the aforementioned methods with the aforementioned vascular PDT. This combination could act bi-directionally—on the one hand, to lead to damage of cancer cells by hypoxia-induced destruction of blood vessels along with activation of the immune response, and on the other hand, to prevent immunosuppressive effects of hypoxia. Nanomicelles composed of modified sericin, tumor-targeting factor V12, and PS IR780, synthesized by Guo et al., also have the ability to activate NLRP3/caspase-1/gasdermine D (GSDMD)-dependent pyroptosis [116]. This study underscores the importance of the level of photosensitizer accumulation in the activation of the immune response. The nanomicelles, due to their enhanced cellular uptake, enabled the delivery of more PS IR780 to cancer cells. The NIR-activated nanomicelles significantly inhibited ATP syntase expression, leading to mitochondrial damage and activation of pyroptosis, which led to dendritic cell maturation. In addition, Kang MWC et al. showed that combined photodynamic and photothermal therapy using gold nanorods with PS chlorin e6 on endogenously formed mouse serum protein coronas has the ability to enhance activation of dendritic cells and macrophages in EMT6 mouse breast cancer. The differences in cellular responses suggest that an equal level of immune response after therapy cannot be expected for different types of cancer cells [117]. This, in turn, may affect the overall efficacy of phototherapy in different types of cancer. Stimulation of DC maturation by PDT with zinc phthalocyanine mediated by extracellular vesicles derived from M1-like macrophages was also achieved by Huis In ‘t Veld RV et al. resulting in a complete (100%) response in MC38 mouse colon adenocarcinoma tumors. To achieve optimal anti-tumor efficacy using extracellular vesicles as a vehicle for PS, both the cell type and their immune status must be taken into account. It is important to note, however, that depending on the cellular origin, these vesicles can also promote tumor growth [118]. Chitosan also has the ability to promote DC maturation by inducing type I interferons [119]. Dendritic cell vaccines may also be promising inducers of the adaptive immune response. This was demonstrated by Redkin TS et al. by creating a vaccine from DCs pulsed with lysates of PDT-treated glioma cells with tetracyanetetra(aryl)porphyrazines I and III [21]. This study suggests that the choice of PS may affect the efficacy of PDT immunogenicity in cancer treatment. The ability to effectively activate the adaptive antitumor response in the treatment of glioblastoma may be crucial to achieving effective therapy for this cancer. This is particularly important given the unsatisfactory results of treatment with current methods and the poor prognosis of patients. Zhang H et al. showed that ALA-PDT-treated dendritic cell vaccine can increase CD4+ and CD8+ T-cell activity in tumors. Mice that were immunized three times at 7-day intervals showed no tumors after the introduction of cancer cells [32]. Such a vaccine generated by ALA-PDT designed by Ji J et al. inhibited the growth of squamous cell carcinoma in mice. In this study, effective protection against tumor growth was observed, indicating the successful initiation of an adaptive immune response [120]. The promising results of the skin cancer study favor testing the efficacy of this strategy in the context of treating other cancers. However, further studies are needed, also examining the possible side effects of this therapy. Work is also currently underway to combine PDT with immune checkpoint inhibitor therapy. The effectiveness of this approach was demonstrated by Lou et al. who enhanced the immune response by combining PDT with an anti-PD1 monoclonal antibody [35]. This study suggests that the combination of PDT with αPD-1 may be effective in enhancing the adaptive immune response. Since an increase in potassium and phosphorus levels was observed, suggesting a benign tumor breakdown syndrome, further studies are needed to optimize doses and treatment protocol before implementing this strategy in therapy. Duan X et al. developed Zn-pyrophosphate nanoparticles with PS pyrolipid that sensitize tumors to checkpoint inhibition by PD-L1 antibodies and, by enhancing tumor-specific T-cell responses, lead to complete eradication of the primary tumor and metastasis. This strategy makes it possible to improve the efficacy of treatment with PD-L1 antibodies, which benefit only a minority of patients whose tumors have been pre-infiltrated by T cells [28]. In addition, this study suggests the possibility of constructing therapies that can be tailored to specific characteristics and tumor types, enabling personalized therapy. Improvements in cytotoxic T-cell activity were achieved by Anand s et al. who, by associating ALA-PDT with the administration of 5-fluorouracil, reduced the presence of cells expressing the immune checkpoint marker PD-1 [121]. This study highlighted the potential of combining chemotherapy with PDT in activating the adaptive immune response. Finally, Hwang HS et al. showed that CD8+ T-cell function induced by PDT combined with peptide vaccination of a tumor-specific TLR5 agonist can be significantly enhanced by PD-1 checkpoint inhibitor therapy [122]. The study confirms the potential of PDT as a component of combination immune therapy, especially in the “cold tumor” melanoma model. Jiang X. et al. showed that improving the effectiveness of the adaptive immune response is led by the combination of PDT and a toll-like receptor agonist in the form of chlorin e6/R848 polymer [123]. Finally, a molecule with PEGylated PS and an indoleamine 2,3-dioxygenase 1 inhibitor designed by Gao et al. suppressed CT26 colon cancer recurrence in mice [124]. Rocha LB et al. showed that PDT with bacteriorhodopsin provides immune memory and inhibits CT26 metastasis [125]. Cheng et al. report on a chimeric peptide they designed for the treatment of malignant melanoma with PpIX and a melanoma antigen-specific peptide capable of inducing cancer cell death and activating an anti-tumor immune response [126].

In conclusion, the studies discussed indicate the potential of various therapeutic combinations of PDT with modalities to achieve synergy in the activation of adaptive immune responses. Some also demonstrate the potential ability of PDT to induce immune memory, which could represent a significant advance in preventing cancer recurrence.

Table 2 presents the recent reports on PDT and activation of adaptive immune response.

## 7. Future Research Directions

Despite the considerable achievements of the research conducted to date, it is necessary to further improve the effectiveness of PDT in activating the adaptive immune response. As mentioned in earlier sections, this presents a number of challenges. These include the suboptimal properties of photosensitizers, the lack of a precisely defined therapeutic regimen with the greatest efficacy, and the immunosuppressive tumor microenvironment. Future research directions should focus on this area. There is a constant need to develop new photosensitizers with optimal properties, such as the ability to selectively accumulate in cancer cells with minimal induced side effects. The ability to activate a specific immune response depends on the type of compound used, so future research should be guided by current experiments in this area, such as the relationship of PS structure, location of accumulation in the cell, and type of cell death induced and its ability to induce an adaptive response. The effectiveness of therapy does not depend only on the properties of the photosensitizer used, so it is also necessary to determine the most favorable therapeutic regimen. It should take into account the optimal incubation time needed for absorption of a given PS, the dedicated laser wavelength, and irradiation time. Future work should also focus on the ability of the immunosuppressive tumor environment to inhibit the adaptive immune response induced by PDT. Previous work indicates that PDT is effective when combined with other forms of therapy in this area. Work is also warranted to add to the current state of knowledge about PDT’s ability to induce activation of the specific response. Its expansion and a precise understanding of this complex mechanism can provide the basis for precise research and the discovery of new strategies to improve the efficacy of PDT. Table 3 presents the summary of the effects of PDT stimulating a specific immune response and their mechanism.

## 8. Conclusions

Cancer is a significant cause of mortality worldwide, and the type of therapy used depends on the anatomical location of the tumor, its type, and degree of progression. One form of therapy used to treat cancers is photodynamic therapy. This article presents recent findings on the activation of the adaptive immune response by reactive oxygen species triggered by photodynamic therapy. The exact mechanism of this process is not yet understood. It has been established that it involves tumor cell death responsible for the release of molecular patterns associated with damage, followed by activation of antigen-presenting cells and induction of tumor-specific T-cell responses. In addition, there is also the involvement of other cells and the production of anti-tumor antibodies. Importantly, this strategy allows for the treatment of metastatic lesions and has the ability to prevent cancer recurrence. Some studies have also shown that it can prevent the development of cancers from other systems and cells. However, the activation of the adaptive immune response by PDT still presents many challenges including the suboptimal properties of photosensitizers, the lack of a precisely defined therapeutic regimen with the greatest efficacy, and the immunosuppressive tumor microenvironment. This article presents evidence that TME has the ability to suppress the immune system through hypoxia, soluble mediators, the extracellular matrix, or the presence of numerous suppressor cells. A precise understanding of the mechanism by which PDT induces an adaptive immune response and the reasons for its failure is essential to improve its anti-tumor efficacy. The effectiveness of PDT can be increased by combining PDT with other forms of therapy. As outlined in this article, there has been a lot of research in this area over the past 10 years. Their results are exciting. The multitude of strategies being tested and their high effectiveness means that an optimal combination of several of them could lead to the end of the cancer problem in at least a few types of cancer. This idea seems particularly interesting in the context of the rapid development of artificial intelligence currently being observed, its ability to analyze information multi-dimensionally, and the increasing reports of its application in medicine. However, it is important to remember that this research is in its early stages and many more observations need to be made for clinical trials and applications in the clinic. In conclusion, photodynamic therapy’s ability to activate the adaptive immune response represents great potential in cancer therapy, and further necessary research may contribute to its effectiveness in cancer treatment.

## Figures and Tables

**Table 1:** Inclusion and exclusion criteria for review.

***Inclusion***
Papers describing adaptive immune response induced by PDT were included
Both review articles and research articles were included
Papers written since 2014 were included
Both in vivo and in vitro studies were included
Papers describing reports on the immune efficacy of PDT were included
Papers describing the reasons for the failure of PDT to activate the adaptive immune response have been included
***Exclusion***
Papers focusing on the innate immune response induced by PDT were excluded
Articles in a language other than English or Polish
Papers written before 2014 were excluded
Papers describing adaptive immune response induced by PDT in the treatment of diseases other than cancer were excluded

**Table 2:** Recent reports on PDT and activation of adaptive immune response.

Authors	Title	Year of Publication	Results
Lou J et al. [35]	Repeated photodynamic therapy mediates the abscopal effect through multiple innate and adaptive immune responses with and without immune checkpoint therapy	2023	The association of PDT with anti-PD1 monoclonal antibody has the ability to enhance the immune system response
Anand S et al. [121]	Combination of 5-Fluorouracil with Photodynamic Therapy: Enhancement of Innate and Adaptive Immune Responses in a Murine Model of Actinic Keratosis	2023	Combining 5FU and PDT with PS protoporphyrin IX may work synergistically, and provide better treatment for squamous cell carcinoma
Jiang X et al. [123]	Nanoscale coordination polymer synergizes photodynamic therapy and toll-like receptor activation for enhanced antigen presentation and antitumor immunity	2023	The association of PDT and toll-like receptor agonist in the form of chlorin e6/R848 polymer has the ability to induce immunogenic death of MC38 colon cancer cells achieving a 50% cure rate and 99.4% inhibition of tumor growth
Redkin TS et al. [21]	Dendritic Cells Pulsed with Tumor Lysates Induced by Tetracyanotetra(aryl)porphyrazines-Based Photodynamic Therapy Effectively Trigger Anti-Tumor Immunity in an Orthotopic Mouse Glioma Model	2023	Dendritic cell vaccines pulsed with the lysates of glioma GL261 cells pre-treated with pz-I-PDT or pz-III-PDT could act as effective inducers of adaptive anti-tumor immunity in an intracranial orthotopic glioma mouse model
Huis In ‘t Veld RV et al. [34]	M1-derived extracellular vesicles enhance photodynamic therapy and promote immunological memory in preclinical models of colon cancer	2022	PDT with PS zinc phthalocyanine mediated by extracellular vesicles derived from M1-like macrophages has the ability to induce a complete MC38 tumor response in mouse models
Guo W et al. [116]	VB12-Sericin-PBLG-IR780 Nanomicelles for Programming Cell Pyroptosis via Photothermal (PTT)/Photodynamic (PDT) Effect-Induced Mitochondrial DNA (mitoDNA) Oxidative Damage	2022	PDT with nanomicelles composed of modified sericin, tumor-targeting agent V12, and PS IR780 has the ability to induce tumor cell pyroptosis and generate anti-tumor immunity
Su X et al. [30]	A Carbonic Anhydrase IX (CAIX)-Anchored Rhenium(I) Photosensitizer Evokes Pyroptosis for Enhanced Anti-Tumor Immunity	2022	PDT with rhenium(I) photosensitizer anchored to carbonic anhydrase IX has the ability to induce pyropotic tumor cell death and stimulate tumor immunogenicity
Zhang et al. [32]	Enhancement of innate and adaptive anti-tumor immunity by serum obtained from vascular photodynamic therapy-cured BALB/c mouse	2021	Vascular PDT (VPDT) has the ability to induce varying degrees of resistance to attack other types of mouse tumor cells
Gao A et al. [124]	Sheddable Prodrug Vesicles Combating Adaptive Immune Resistance for Improved Photodynamic Immunotherapy of Cancer	2020	PDT with PEGylated PS and indoleamine 2,3-dioxygenase inhibitor 1 has the ability to suppress CT26 colon cancer recurrence
Hwang HS et al. [122]	Combination of Photodynamic Therapy and a Flagellin-Adjuvanted Cancer Vaccine Potentiated the Anti-PD-1-Mediated Melanoma Suppression	2020	PDT in combination with peptide vaccination of a tumor-specific TLR5 agonist can be enhanced by association with a PD-1 checkpoint inhibitor
MWC et al. [117]	Innate immune activation by conditioned medium of cancer cells following combined phototherapy with photosensitizer-loaded gold nanorods	2020	Combination of PDT and photothermal therapy using gold nanorods with PS chlorin e6 on endogenously formed mouse serum protein coronas has the ability to enhance activation of dendritic cells and macrophages in breast cancer EMT6
Im S et al. [47]	Hypoxia-Triggered Transforming Immunomodulator for Cancer Immunotherapy via Photodynamically Enhanced Antigen Presentation of Dendritic Cell	2019	PDT can be used in dendritic cell-based immunotherapy
Cheng et al. [126]	A Self-Delivery Chimeric Peptide for Photodynamic Therapy Amplified Immunotherapy	2019	PDT with the chimeric peptide PpIX-PEG8 -KVPRNQDWL has the ability to induce an anti-tumor immune response in the treatment of malignant melanoma
Zhang H et al. [32]	Antitumor Effects of DC Vaccine With ALA-PDT-Induced Immunogenic Apoptotic Cells for Skin Squamous Cell Carcinoma in Mice	2018	PDT dendritic cell vaccination is an effective prophylactic therapy for squamous cell carcinoma
Oh DS. et al. [82]	Intratumoral depletion of regulatory T cells using CD25-targeted photodynamic therapy in a mouse melanoma model induces antitumoral immune responses	2017	PDT targeting tumor-associated regulatory T cells can specifically modulate the tumor microenvironment and may be used as a new technique for cancer immunotherapy
Duan X et al. [28]	Photodynamic Therapy Mediated by Nontoxic Core-Shell Nanoparticles Synergizes with Immune Checkpoint Blockade To Elicit Antitumor Immunity and Antimetastatic Effect on Breast Cancer	2016	PDT of Zn-pyrophosphate nanoparticles with PS pyrolipid has the ability to sensitize tumors to checkpoint inhibition by PD-L1 antibodies and lead to complete eradication of primary tumor and metastasis
Shams et al. [114]	Development of photodynamic therapy regimens that control primary tumor growth and inhibit secondary disease	2015	PDT can be an effective adjuvant for therapies that do not stimulate the host’s anti-tumor immune response
Ji J et al. [120]	DC vaccine generated by ALA-PDT-induced immunogenic apoptotic cells for skin squamous cell carcinoma	2015	ALA-PDT-DC vaccine has ability to inhibit the growth of skin squamous cell carcinoma
Rocha LB et al. [125]	Elimination of primary tumors and control of metastasis with rationally designed bacteriochlorin photodynamic therapy regimens	2015	PDT can be an effective adjuvant for therapies that do not stimulate the host’s anti-tumor immune response
Wachowska et al. [7]	5-Aza-2′-deoxycytidine potentiates antitumor immune response induced by photodynamic therapy	2014	Induction of expression of silenced tumor P1A antigen by 5-Aza-2′-deoxycytidine may enhance activation of PDT-induced adaptive immune response

**Table 3:** Effects of PDT stimulating specific immune response and their mechanism.

Effect	Mechanism
DAMP release	Generation of damage and cell death by produced reactive oxygen species
Increase the ability of dendritic cells to present antigens	Stimulation of dendritic cell maturation by released DAMPs
	Increased expression of MHC II on the surface of DCs
	Promotion of DC maturation by released tumor lysates
Increased CD8+ T-cell response	Increase in activation of CD8+ T cells due to increased antigen cross-presentation
	Increased incidence of CD8+ T cells in distal non-irradiated lymph nodes draining the tumor
Induction of anti-tumor response of other cells	Production of tumor-specific antibodies that stimulate engulfment of cancer cells by macrophages and activate neutrophil antibody-dependent cytotoxicity
